# Seasonal Trophic Niche Shift and Cascading Effect of a Generalist Predator Fish

**DOI:** 10.1371/journal.pone.0049691

**Published:** 2012-12-14

**Authors:** Jun Xu, Zhourui Wen, Zhijun Gong, Min Zhang, Ping Xie, Lars-Anders Hansson

**Affiliations:** 1 Donghu Experimental Station of Lake Ecosystems, State Key Laboratory of Freshwater Ecology and Biotechnology of China, Institute of Hydrobiology, Chinese Academy of Sciences, Wuhan, Hubei, People's Republic of China; 2 Hubei Fishery Science Institute, Wuhan, Hubei, People's Republic of China; 3 Nanjing Institute of Geography and Limnology, Chinese Academy of Science, Nanjing, Jiangsu, People's Republic of China; 4 College of Fisheries, Huazhong Agricultural University, Wuhan, Hubei, People's Republic of China; 5 Institute of Biology/Aquatic Ecology, Ecology Building, Lund University, Lund, Sweden; University of Otago, New Zealand

## Abstract

Few studies have examined how foraging niche shift of a predator over time cascade down to local prey communities. Here we examine patterns of temporal foraging niche shifts of a generalist predator (yellow catfish, *Pelteobagrus fulvidraco*) and the abundance of prey communities in a subtropical lake. We predicted that the nature of these interactions would have implications for patterns in diet shifts and growth of the predator. Our results show significant decreases in planktivory and benthivory from late spring to summer and autumn, whereas piscivory increased significantly from mid-summer until late autumn and also increased steadily with predator body length. The temporal dynamics in predator/prey ratios indicate that the predation pressure on zooplankton and zoobenthos decreased when the predation pressure on the prey fish and shrimps was high. Yellow catfish adjusted their foraging strategies to temporal changes in food availability, which is in agreement with optimal foraging theory. Meanwhile the decrease in planktivory and benthivory of yellow catfish enabled primary consumers, such as zooplankton and benthic invertebrates, to develop under low grazing pressure via trophic cascading effects in the local food web. Thus, yellow catfish shifts its foraging niche to intermediate consumers in the food web to benefit the energetic demand on growth and reproduction during summer, which in turn indirectly facilitate the primary consumers. In complex food webs, trophic interactions are usually expected to reduce the strength and penetrance of trophic cascades. However, our study demonstrates strong associations between foraging niche of piscivorous fish and abundance of prey. This relationship appeared to be an important factor in producing top-down effects on both benthic and planktonic food webs.

## Introduction

Foraging behavior is strongly modulated by spatio-temporal variations in food availability [1], [2], and coherence between predators and their prey is common in a variety of ecosystems, because predators must continuously track changing prey patterns and respond to complex heterogeneities in space and time [3], [4]. Predators will shift to alternative prey when the density of their preferred food is low and it is argued that alternative food may play an important role in promoting persistency and also stabilizing the equilibrium of predator-prey systems [2].

Size has a predominant influence on an animal's energetic requirements and thus impose important constraints on animal's resource exploitation and interaction with other species [5], [6]. Given that resource utilization abilities are generally related to body size, many species will undergo extensive ontogenetic shifts in foraging strategies, which can be viewed as navigating a landscape of foraging niches [5]. Understanding such patterns will help us to predict the trajectory a species will take at the various life history stages and the ecological consequences that this trajectory may have.

A growing interest has emerged regarding the response of foraging predators to prey dynamics [5], [7], [8], [9]. However, most studies are theoretical and experimental studies, as well as the complex interactions and the corresponding responses in the natural environments, are therefore still less understood. Given that utilization of prey are generally related to both body size and resource availability, predators will undergo extensive foraging niche shifts in the natural environment, which in turn will shape the structure of communities in which these interactions are imbedded [10]. Thus, empirical studies are still needed to understand predator-prey relationships in order to improve our understanding of natural communities, but also to promote correct decision-making for the management of exploited populations and the conservation of species [1], [3].

The primary goal of this study was to investigate the seasonal dynamics in foraging niches of a generalist predatory fish by using a stable isotope mixing model in a subtropical lake. We first tested whether the foraging niches changed seasonally, and then compared the relationships between prey abundance and the foraging niches to assess the evidence for niche partitioning dependence on resource availability. Finally, we analyzed the size-related niche shift and evaluated its influence on the seasonal niche shift as a covariant, and also studied the ecological impact of the foraging niche shift on the local community.

## Materials and Methods

### Ethics Statement

Ethical approval was given by the ethical committee at Institute of Hydrobiology, Chinese Academy of Science. In the current study, the use of tissue material from animals killed is a part of routine commercial fishery production. The commercial fishery complies with the local fishery law in Jiangsu Province. No specific permits were required for the described field studies. The location studied is not privately-owned or protected in any way and the field studies did not involve endangered or protected species.

### Sampling

The study was conducted on the eastern (predominantly macrophyte-covered) side of Gonghu Bay (31°25′N; 120°18′E), Lake Taihu China, between April and November 2005. Three sites were randomly selected as replicates and were separated from each other by at least 3000 m. The dominant predatory fish is yellow catfish (*Pelteobagrus fulvidraco*, Richardson, 1846), whose prey organisms are zooplankton, benthic macroinvertebrates, freshwater shrimps and small fishes [11], [12]. Yellow catfish and its prey were censused at these sites once every month during the investigation period ( April to November 2005). Fish were sampled using trawlnets (each 2 m×2 m, mesh size = 7.0 mm, warp length = 3 m) towed at each sampling site with a net speed of approximately 4000 m per hour. All fish were sorted aboard the vessel and were counted and weighted. The freshwater shrimps (*Exopalaemon modestus* and *Macrobrachium nipponensis*), and prey fish, such as *Pseudorasbora spp.*, *Rhinogobius spp.*, and *Odontobutis spp.*, were also counted and weighted. This size and type of fish are the main prey items of yellow catfish in this lake [11], [12]. Benthic macroinvertebrates, including the snail *Bellamya aeruginosa*, and the filter-feeding mussel *Cristaria plicata* and *Anodonta woodiana woodiana*, were collected.

Abundance of zooplankton and benthic invertebrates were also assessed in each site. Zooplankton (cladoceran and copepods) were collected using a plankton net (mesh size 112 µm) by filtering 10 L lake water into acid washed PVC bottles (50 ml) and preserved with Lugol solution prior to laboratory identification. Benthic macroinvertebrates were sampled by collecting surface sediments using an Ekman grab (1/40 m^2^), washed with a hand net (mesh size 1 mm) and preserved with 95% ethanol for laboratory identification.

All individuals of yellow catfish, at least five individuals of prey fish and freshwater shrimps of yellow catfish, grazing snail (*B. aeruginosa*) and filter-feeding mussels (*C. plicata* and *A. woodiana woodiana*) were collected per site for stable isotope analysis. Samples were placed on ice and transported to laboratory. For fishes and shrimps, white muscle tissues were dissected in the laboratory. These tissues are representative of the overall stable isotope signature in fish (Hesslein et al. 1993). A sample of foot muscle tissue of molluscs was in the laboratory taken for stable isotope analysis since the shell material is enriched in ^13^C and does not reflect what is actually assimilated by the snail (Mitchell et al., 1996). Muscle samples were oven-dried to a constant weight at 60°C and then ground to a fine homogeneous powder with a mortar and a pestle. The mortar and pestle were acid-washed and dried to prevent cross-contamination between samples. The powdered samples were kept in acid-washed glass tubes and sealed in desiccators with silica gel for future analysis [13].

### Analysis of stable isotopes

The δ^13^C and δ^15^N values were generated after analysis with a Delta Plus (Finnigan, Bremen, Germany) continuous-flow isotope ratio mass spectrometer coupled to a Carlo Erba NA2500 elemental analyzer (Carlo Erba Reagenti, Milan, Italy). Stable isotope ratios were expressed as parts per thousand (‰) deviation from the international standards according to the equation: δX = [(Rsample/Rstandard)−1]×1000, where X is ^15^N or ^13^C and R is the corresponding ratio ^15^N/^14^N or ^13^C/^12^C. δ is the measure of heavy to light isotope in the sample, whereby higher δ values denote a greater proportion of the heavy isotope. The standard for nitrogen was atmospheric nitrogen and that for carbon was Vienna Pee Dee belemnite. The reference material for δ^15^N was ammonium sulfate (IAEA-USGS25), and that for δ^13^C was carbonatite (IAEA–NBS18), supplied by the U.S. Geological Service (Denver, Colombia, USA) and certified by International Atomic Energy Agency (Vienna, Austria). On a daily basis, an internal working standard, urea (δ^15^N = −1.53‰; δ^13^C = −49.44‰) was employed. Twenty percent of the samples were run in duplicate; the average standard errors of replicate measurements for δ^13^C and δ^15^N were both less than 0.3‰.

### Trophic niches calculation

According to the previous gut content analysis [11], [12] (See also Table S1 and Table S2), dominant food items of yellow catfish were classified into three guilds: zooplankton, benthic primary invertebrates, and shrimps and fishes, in order to effectively calculate the feasible contribution to each individual of yellow catfish. Benthic primary consumers included animals such as Oligochaeta, Chironomidae, and snails. We estimated planktivory, benthivory, and piscivory, defined as the estimated reliance on planktonic and benthic primary consumer resources and carnivorous resources of intermediate consumers in the food web, using δ^13^C and δ^15^N data and the SIAR (Stable Isotope Analysis in R) software (Version 2.13.1). SIAR is based on a Bayesian approach that estimates possible distributions of resource contributions to a consumer diet by accounting for all uncertainties of the input data [14]. The effects of differing isotopic compositions over time were eliminated by this calculation from the matrices of stable isotopes of end members. To account for trophic fractionation we used fractionation factors and uncertainties of 0.4±1.3‰ for δ^13^C and 3.4±1.0‰ for δ^15^N [15]. For running the mixing models, the appropriate number of iterations (up to 5×10^5^) was chosen according to SIAR's convergence diagnostic. The procedure then provided information about the range and distribution of possible source contributions. From the resulting up to 30 000 dietary proportions, we selected the maximum feasible contribution of food sources to each individual from the top distribution in histograms. The sum of combinations of each source contribution was 100%.

With respect to this stable isotope methodology, the relatively slow stable isotope turnover in yellow catfish compared to their planktonic and benthic primary consumer resources is a potential concern. We thus used stable isotope data of the grazing snail, *B. aeruginosa*, and the filter-feeding mussels, *C. plicata* and *A. woodiana woodiana*, as the end members for planktivory and benthivory calculations in SIAR. These data were used as estimates of niche shifts between planktonic and benthic primary consumer resources [15], and the results in the mixing model could then reduce the bias caused by stable isotope values of zooplankton and small benthic invertebrates reflecting their feeding on a shorter time period than yellow catfish. For the end members of piscivory calculations in SIAR, we pooled the stable isotope values of the potential carnivorous resources of intermediated consumers in the current food web, including freshwater shrimps, *Exopalaemon modestus* and *Macrobrachium nipponensis*, and prey fish (*Pseudorasbora spp.*, *Rhinogobius spp.*, and *Odontobutis spp.*).

We calculated the predator/prey ratios (PPR) between the predator, yellow catfish, and its prey, zooplankton (PPR_z_), benthic invertebrates (PPR_b_), prey fishes and shrimps (PPR_f_), respectively.

### Statistical analysis

We used analysis of variance (ANOVA) for the comparisons on δ^13^C (and δ^15^N) of end-members of prey sources to test the utility of stable isotope analysis for determining the energy base of the predator fish in time. Type of end-members is used as a fixed factor and time as a random factor. If δ^13^C and δ^15^N of end-members of prey sources are not distinct, the mixing model (SIAR) is not appropriate. Significance levels were α = 0.05. Bonferroni post-hoc test were used to compare among the prey sources.

We tested whether planktivory, benthivory, and piscivory of yellow catfish varied over time using one way ANOVA, with date as a fixed factor and site as a random factor. A non-parametric smoothed curve (LOWESS) was used to search for functional relationships to see if there were any functional change in planktivory, benthivory, and piscivory with the size of yellow catfish.

The relationships between size of yellow catfish and their foraging niches (including planktivory, benthivory, and piscivory) was analyzed with one-way analysis of covariance (ANCOVA) with date as factor, planktivory, benthivory, and piscivory as variables, respectively, and body size as covariate.

The predator-prey ratio was calculated by dividing the total biomass of predator individuals with the total biomass of zooplankton, benthic invertebrates, and forage shrimps and fishes, respectively. All the data were log(1+x) transformed to improve homogeny in the dataset. To further test whether each type of predator-prey ratios differed over time, we used ANOVA with date as a fixed factor. We tested the relationships between biomass of the predator and its prey using linear regression analysis. We also tested the relationship between monthly mean foraging niche indices (including planktivory, benthivory, and piscivory) and each corresponding predator-prey ratio using linear regression analysis, with foraging niches as the explanatory variables and predator-prey ratios as response variables. Seasonal variation in PPR between yellow catfish and its prey was tested using ANOVA.

To separate the combining effects of growth and prey availability, we carried out a progress of statistics. We first extracted trophic niche residuals after the LOWESS analysis (to remove the effects of the predator growth), including planktivory, benthivory, piscivory residuals. We then used Pearson correlation analysis between the residuals and seasonal variations of the prey biomass. This two step is similar to partial correlation analysis. In probability theory and statistics, partial correlation measures the degree of association between two random variables, with the effect of a set of controlling random variables removed. All Statistical analyses were performed with R 2.13.0 [16].

## Results

δ^13^C and δ^15^N of baseline animals showed significant differences by the types of end-members (*F*_2,96_ = 303.2, *p*<0.001, Table S3). In different end-members, δ^13^C (or δ^15^N) of the planktonic food sources (zooplankton and mussels), the benthic food sources (macrozoobenthos and snails), and the piscivorous food sources (shrimps and small fishes) were all distinct during the study (all *p*<0.001). Therefore, the mixing model was applied to the predator fish in different end-members.

**Figure 1:**
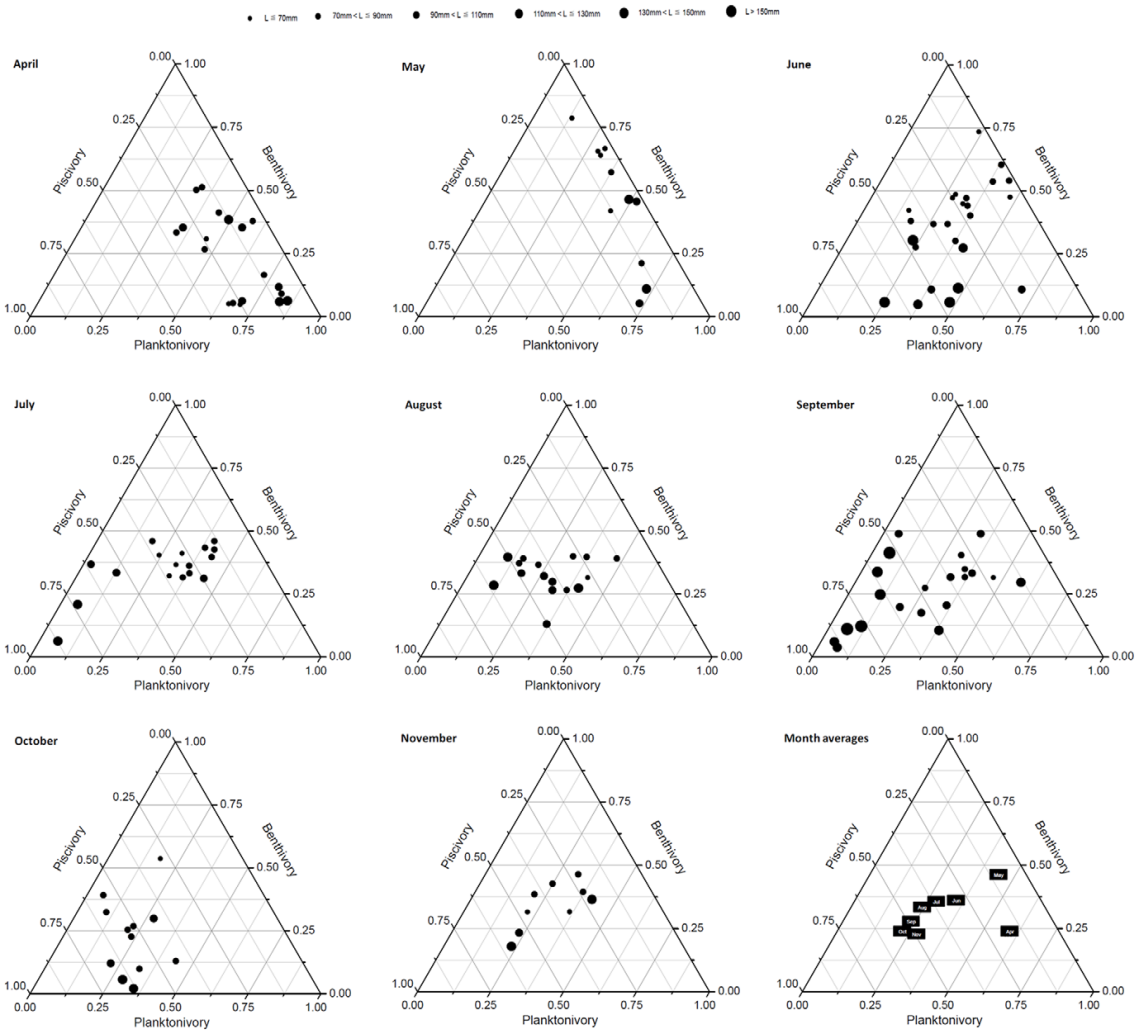
Ternary diagram visualizing the seasonal dynamics of foraging niches calculated from a stable isotope mixing model. Notice that the diagram has three axes with piscivory at the left, benthivory at the right, and planktivory at the bottom. Any point that plots anywhere on one of the side lines, or within the triangle, represents a foraging niche composed of mixed end members. The end members in this isotope mixing model is intermediate consumers (small forage fishes and shrimps) and planktonic and benthic primary consumers (snails and mussels), respectively, for calculation of piscivory, benthivory and planktivory.

The seasonal dynamics in foraging niche (piscivory, benthivory and planktivory) calculated from the stable isotope mixing model is visualized in the ternary diagram (Fig. 1). In April, the percentage of benthivory and planktivory was relatively high, but changed to a high percentage of piscivory later in the season. The trajectory of the foraging niche shift of yellow catfish during the growing season was periodic to some extent.

**Figure 2:**
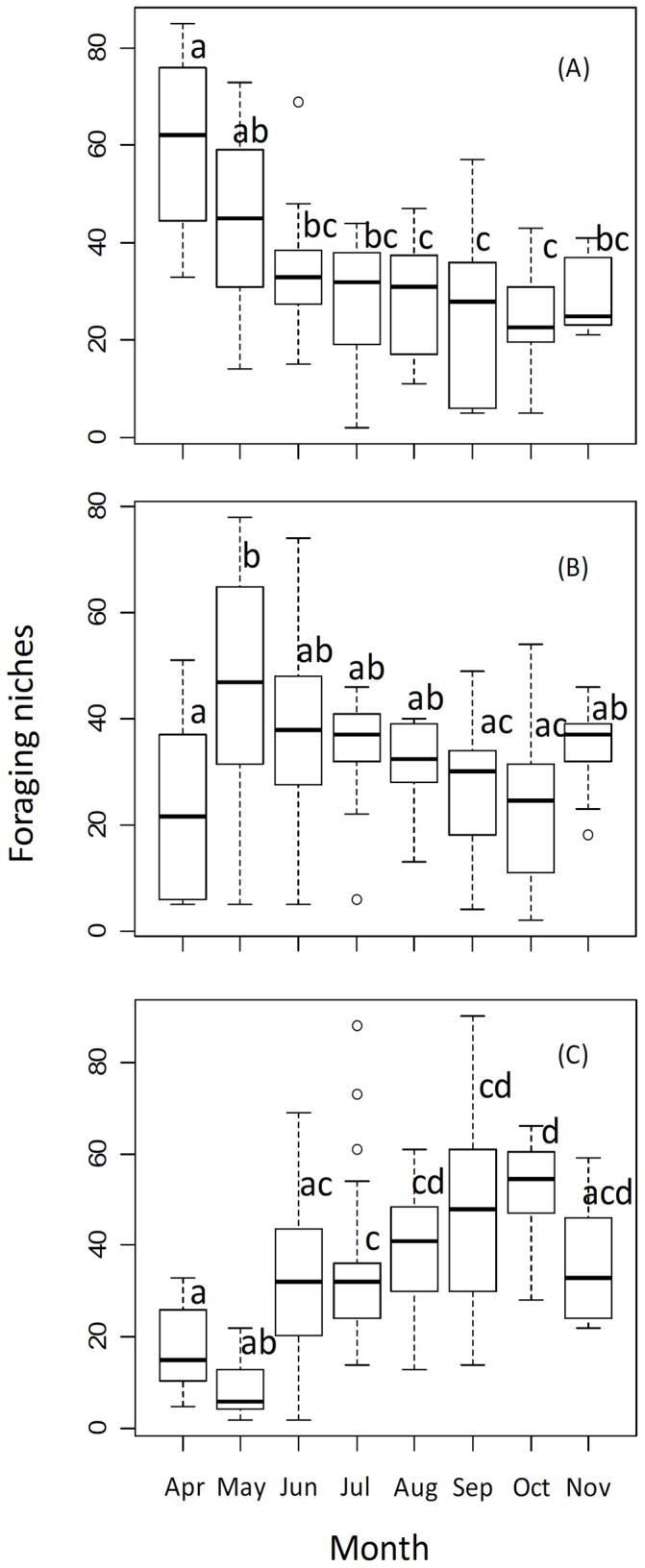
Box-and-whisker plots of seasonal variation in foraging niches of yellow catfish, including planktivory (A), benthivory (B) and piscivory (C). Upper and lower ends of boxes represent 75^th^ and 25^th^ percentiles. Whiskers represent 75^th^ and 25^th^ percentiles and open circles, outliners. The mean is depicted with a solid line. ANOVA indicated there were significant seasonal changes in planktivory (*F*_7,125_ = 13.055, *P*<0.001), benthivory (*F*_7,125_ = 3.7291, *P*<0.005) and piscivory (*F*_7,125_ = 12.307, *P*<0.005). Different letters show the statistically significant differences indicated by a post hoc Tukey's test.

Our data suggest that there were significant seasonal changes in planktivory (*F*_7,125_ = 13.055, *P*<0.001), benthivory (*F*_7,125_ = 3.729, *P*<0.005) and piscivory (*F*_7,125_ = 12.307, *P*<0.005). Specifically, planktivory of yellow catfish decreased during the season, whereas benthivory increased in spring and then decreased from late spring to autumn. Piscivory showed low values in spring, but increased significantly from mid-summer to late autumn, and, accordingly, both benthivory and planktivory decreased during the same period (Fig. 2)

ANCOVAs of the foraging niche (including planktivory, benthivory, and piscivory) of yellow catfish suggest the interaction effects of season and size on the foraging niches (for planktivory, *F*_7,117_ = 5.532, *P*<0.001); for benthivory, *F*_7,125_ = 2.731, *P*<0.05; and for piscivory, *F*_7,125_ = 4.124, *P*<0.001). The results indicate that the strength of size related foraging niche shifts varied among the sampling dates. Moreover, there were differences for benthivory and piscivory as indicated by the slopes of the linear regressions (*F*_1,117_ = 44.837, *P*<0.001 and *F*_1,117_ = 52.285, *P*<0.001, respectively).

**Figure 3:**
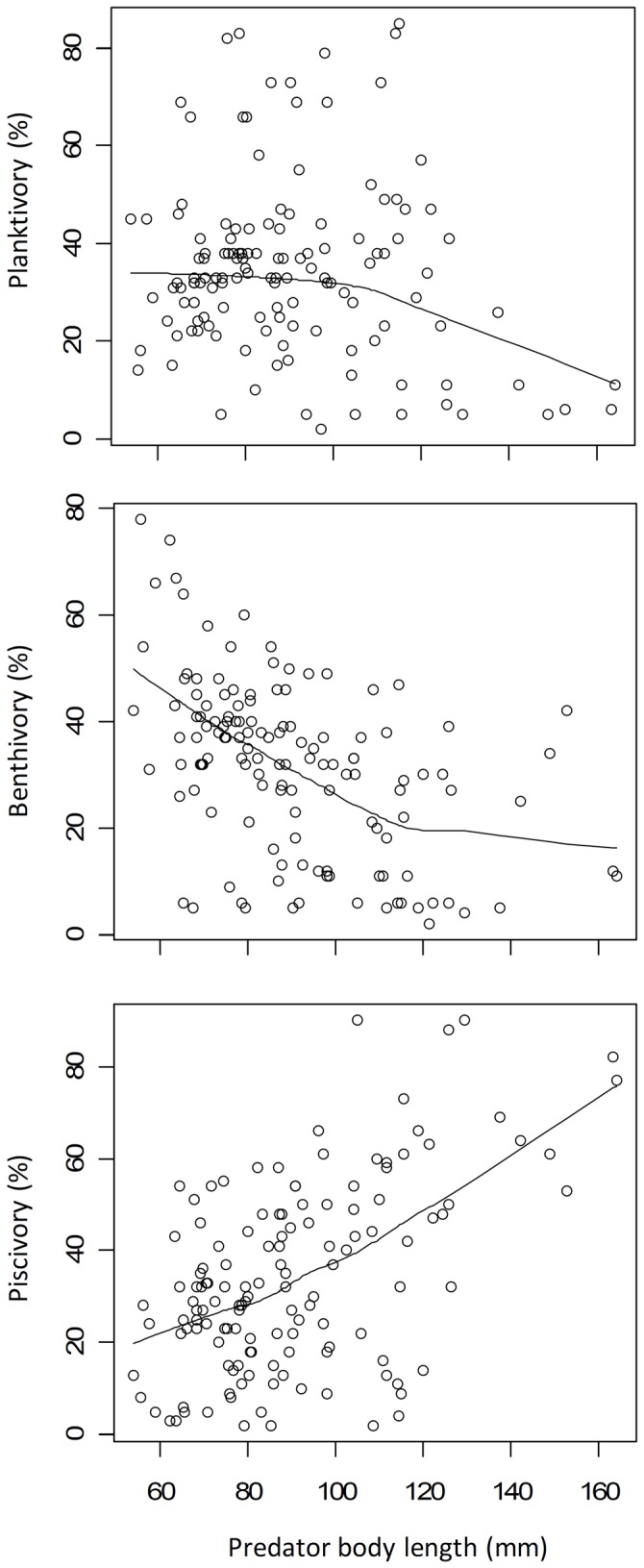
Trends in foraging niches of yellow catfish along size gradient, including planktivory, benthivory and piscivory. LOWESS smoothing curves (the smoother span was set at 1) have been fitted to size series of yellow catfish. On a percentage basis, planktonivory of yellow catfish remained stable before the size of approximate 120 mm and decreased to a low level, and benthivory decreased and reached to a steady low level after the size of approximate 120 cm, while piscivory has been increasing steadily over the range of body length.

LOWESS smoothing curves (the smoother span was set to 1) were fitted to size of yellow catfish to identify ontogenetic trends in foraging niches of yellow catfish (Fig. 3). Planktivory of yellow catfish remained stable up to a size of approximately 120 mm and then decreased to a low level, whereas benthivory decreased and reached to a steady, low level above a size of 120 mm. On the contrary, piscivory increased steadily throughout the range in body length.

Pearson correlation analysis between trophic niche residuals after the LOWESS analysis and the prey biomass revealed that planktivory and piscivory of yellow catfish were significantly negatively (r = −0.791, p<0.01) and positively (r = 0.683, p<0.05) correlated with abundance of prey fishes and shrimps, respectively (see Table S4). Moreover, a marginally significant correlation (r = −0.425, p<0.10) between planktivory of yellow catfish and zooplankton biomass was also found in this study.

**Figure 4:**
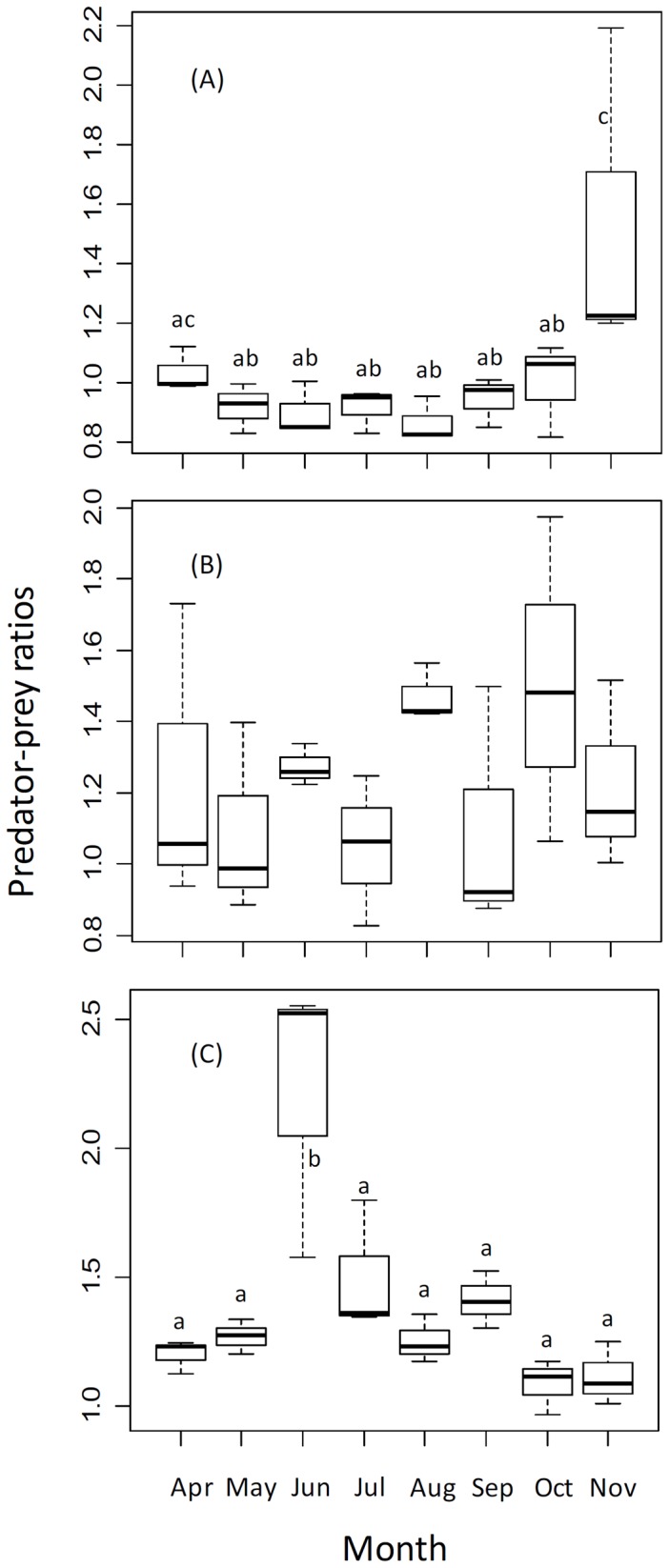
Box-and-whisker plots of seasonal variation in predator/prey ratios between the predator, yellow catfish, and its prey, zooplankton (PPR_z_, A), benthic invertebrates (PPR_b_, B), forage fishes and shrimps (PPR_f_, C), respectively. Upper and lower ends of boxes represent 75^th^ and 25^th^ percentiles. Whiskers represent 75^th^ and 25^th^ percentiles and open circles, outliners. The mean is depicted with a solid line. ANOVA indicated there were significant seasonal changes in PPR_z_ (*F*_7,16_ = 2.9826, *P*<0.05) and PPR_f_ (*F*_7,125_ = 7.4025, *P*<0.001), but not PPR_b_ (*F*_7,125_ = 0.9975, *P* = 0.467). Different letters show the statistically significant differences indicated by a post hoc Tukey's test.

The results show a significant seasonal change in PPR_z_ (*F*_7,16_ = 2.9826, *P*<0.05) and PPR_f_ (*F*_7,125_ = 7.4025, *P*<0.001), but not PPR_b_ (*F*_7,125_ = 0.9975, *P* = 0.467). A decrease in PPR_z_ was observed at the beginning of the season and increased in late autumn; PPR_f_ showed significant increase in the late spring and then decreased from mid-summer to late autumn. The statistically significant differences indicated by a post hoc Tukey's test are summarized in Fig. 4.

**Figure 5:**
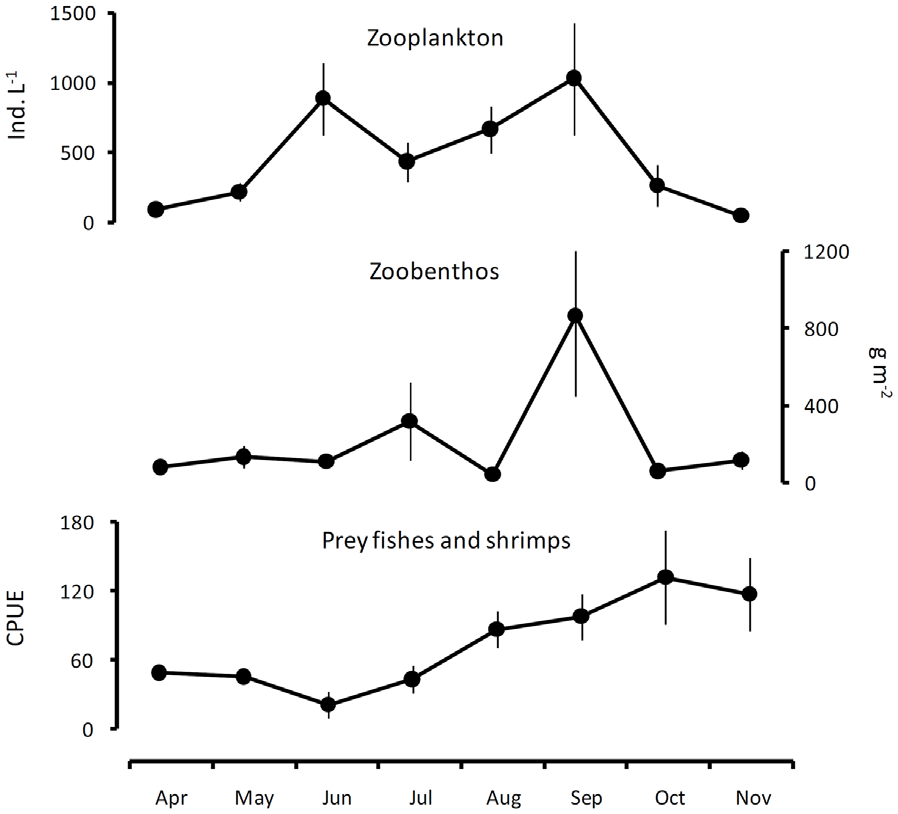
Seasonal abundance of prey of yellow catfish. Mean values are plotted with standard errors integrated over all sampling sizes.

No significant relationships were found between the monthly mean values of foraging niche indices (including planktivory, benthivory, and piscivory) and each corresponding predator-prey ratio. The seasonal variation in abundance of prey items for yellow catfish show peaks of zooplankton and zoobenthos in September and of prey fish and shrimp in October (Fig. 5). Significant positive relationships were found between biomass of the predator and its prey, zooplankton and benthic invertebrates (*R* ^2^ = 0.8431, *F*_1,6_ = 32.24, *P*<0.001 and *R* ^2^ = 0.6621, *F*_1,6_ = 11.76, *P*<0.05, respectively).

## Discussion

Foraging niche shifts play an important role in shaping the feeding strategies of predators in all habitats. In aquatic ecosystem, prey organism abundance vary by several orders of magnitude over time, suggesting a considerable plasticity in feeding niche choice by fish predators. Fishes also show flexible feeding habits and sometimes undergo diet shifts that deviate from their presumed food sources [17], e.g., shifts between the benthic and planktonic food webs [18], [19]. Among fish, for instance, ontogenetic changes in resource use is a common feature during their life cycle [5], [20]. Diet shifts of fishes are usually due to migration, size-related morphological constraints, habitat use and other reasons [21], [22], and thus consequently affect species interactions and community structure [5]. On the other hand, diet shifts can also be expressed as changes in food source utilization or trophic levels of fish [19], which integrate changes in population dynamics, prey availability and predator–prey interactions. Most predatory fish species have to switch from small prey to fish diet during their first or second year of life [23]. With respect to the predator fish in focus for this study, the yellow catfish, it ranges from 50 mm to 170 mm in length, which are mainly 1^+^–2^+^ year old individuals [11], [12]. In such a growing stage, both primary consumers, such as zooplankton and benthic invertebrates, and small fishes and shrimps may be incorporated in its diet [11]. As shown in our study, yellow catfish increased its piscivory during the season, whereas the decrease in planktivory and benthivory enabled primary consumers, such as zooplankton and benthic invertebrates, to develop under low predation pressure. Thus, yellow catfish shifts its foraging niche towards intermediate consumers in the food web during the season, which in turn indirectly facilitates the primary consumers.

### Resource availability and seasonal trophic niche shift

Seasonal heterogeneity of prey abundance and availability plays a role in determining relative prey profitability, because the distribution of prey has a strong effect on energetic gains and costs of foraging [24], foraging success, and overall predator performance [25]. In communities where predators and prey have coexisted for long time, such as those in the current study, predators often respond to prey by developing specific foraging strategies to track the rapidly changing prey patterns in time and space [26], [27]. During the initial period of the summer season, densities of zooplankton and benthic invertebrates decreased due to the intense predation pressure. In such situations, predators are in need for improved hunting efficiency which leads to greater suppressive effect on the large size preys. Our results show that the changes in the foraging niches of yellow catfish imposed intense population suppression on prey fishes late in the season, mirrored in a high predator/prey fishes ratio.

### Size-related foraging niche adaptation

Trends in foraging niches of yellow catfish along a size gradient, including planktivory, benthivory and piscivory were found in this study. On a percentage basis, planktonivory of yellow catfish was high and constant before the size of approximately 120 mm, after which it decreased to a low level. Similarly, benthivory decreased with size and reached a steady low level at sizes above 120 mm, while piscivory increased steadily with body length. Most aquatic food web studies associated with body size reveal that larger fish have higher trophic positions because they are able to consume larger prey [28], [29]. Changes in morphology, behavior, habitat, and distribution during the ontogeny have been suggested to be associated with changes in feeding strategies of organisms [30], [31]. For yellow catfish, one potential explanation for the size-related foraging niche adaptation is the efficient use of prey with different body size, such as zooplankton, zoobenthos and secondary consumers, for example decapods and small fishes. In the current study area, zoobenthos weights were, on average, more than two orders of magnitude larger than zooplankton weight [32]. Furthermore, secondary consumers prey, such as decapods and small fishes, had average weights more than two to three orders of magnitude larger than zoobenthos. The existence of large-sized prey translates into greater foraging profitability for *P. fulvidraco* feeding on secondary consumers relative to zooplankton and zoobenthos, although prey abundances and availability undoubtedly also play a role in determining relative prey profitability as discussed above. This energetic benefit of feeding on large prey is consistent with studies based on optimal foraging theory of of other fish species [5]. Combining the analysis of fish stomach contents and the stable isotope analysis has demonstrated that body size of predators and their prey are significantly correlated with each other, but not with trophic level [33].

### Changes of top-down effects associated with foraging niche shift

Empirical studies from a variety of systems indicate that trophic cascades are widespread, although many factors regulate their occurrence [34]. In natural food webs, trophic interactions are complex, which could reduce the strength and penetrance of trophic cascades [35]. However, strong trophic interactions can lead to trophic cascades in complex systems and further influence the properties of the systems [34], [36]. Moreover, also large-scale environmental changes, such as climate change and brownification, may affect the strength of the feeding links in aquatic ecosytesms [37].

Yellow catfish is a functionally dominant species because it is the only top predator species present in the study area during our sampling period. Prey of yellow catfish, including small fishes and shrimps, commonly feeding on algae, zooplankton and benthic invertebrates, and therefore overlap in food choice with yellow catfish [11], [12], [38]. Foraging niche shift by the top predator thus affect the functional response of the prey and its food. As the predator/prey ratios indicate, the foraging niche of yellow catfish shifted from zooplankton and zoobenthos in spring to secondary consumer preys in summer and resulted in a compensatory increase in abundances of zooplankton and zoobenthos in summer. The consequence was that total planktivory and benthivory actually decreased as piscivory of yellow catfish increased over the study period, which was mirrored in a predation-driven change in the abundance of fish prey. The increased predatory pressure on secondary consumers from yellow catfish thus facilitated for primary consumers both in planktonic and benthic food webs through top-down control. However, seasonal zooplankton dynamics, and the mechanisms driving their variability, are highly susceptible to changes in environmental conditions. For example, other filter-feeding and highly efficient zooplanktivorous fish species may also generate high predation pressure on zooplankton, and seasonal dynamics in nutrent dynamics may cause seasonal changes in phytoplankton productivity and standing stock, which affects zooplankton foraging and subsequent biomass development [39], [40]. Although our results cannot exclude alternative bottom-up and top-down processes affecting the dynamics of primary consumers, the statistical results suggested that planktivory and piscivory of yellow catfish were significantly negatively and positively correlated with abundance of prey fishes and shrimps, respectively. In addition, the marginally significant correlation of planktivory of yellow catfish with zooplankton biomass suggests that zooplankton abundance was governed partially by the predation from yellow catfish. However, these correlation does not necessarily implies cause-effect. For example, no relationship was found between invertebrate prey abundance and benthivory of yellow catfish, and thus the low number of yellow catfish in this lake might not act alone to control the changes in prey abundance.

In freshwater lakes, top-down effects from piscivorous fish can reduce the predation pressure on zooplankton by preying upon planktivorous fish, with cascading effects on the phytoplankton [41]. In addition, consumption of benthic prey by fish is thought to subsidize top-down control in the planktonic food web in some systems [42]. In the present study, the increase in piscivory by yellow catfish leads to another important consequence, the uncoupling of the predator-prey between the yellow catfish and the primary consumers (zooplankton and zoobenthos). It has been suggested that the top-down effect is generally weak in lakes situated in warmer regions around the world [43], because the high abundance, small size and multiple reproductive events of several native omnivorous fishes result in a high predation pressure on primary consumers year around [44], [45]. However, we show here that despite Lake Taihu is a sub-tropical lakepredatory catfish control the abundance of the intermediate consumers, such as prey fishes and shrimps, which allows the development of primary consumers through seasonal niche shifts.

Yellow catfish is an important commercial fish species in Lake Taihu and the preferred catchable size is typically larger than 120 mm, i.e. in the piscivorous stage. Continued fishing on yellow catfish from September and the following months reduces the portion of large size classes resulting in less predation on intermediate consumers, such as prey fish and shrimps, which reach maximum abundances at the end of the growing season. The consequence is a recovery of intense predation on zooplankton and zoobenthos, leading to a the rapid decrease of their abundances and, consequently, intesified algal blooms.

In summary, yellow catfish, a generalist predator, is capable of seasonally responding to local changes in prey abundance and availability. These response patterns were consistent with foraging niche shifts of yellow catfish as indicated by stable isotope signatures. The foraging niche shift of yellow catfish can be regarded as an adaptation to match prey abundance and availability at a temporal scale. Size-related foraging niche shifts of this species are also an important adaptation to resource use during its ontogeny. The season- and size-specific nature of these interactions is of great importance for the local structure and function of the ecosystem, and results in the seasonal dynamics of trophic cascades. Further studies of the impact of niche shift on resource and community dynamics in this eutrophic lake are necessary to better understand this complicated issue and to provide a better framework for management and conservation.

## Supporting Information

Table S1 A Occurrence of food items in yellow catfish of different size in Lake Taihu. B. Dietary composition of yellow catfish in Lake Taihu.
(DOCX)

Table S2 **Seasonal variation in stable isotopic composition, weight and length of yellow catfish.**
(DOCX)

Table S3 **Seasonal variation in stable isotopic composition of yellow catfish prey used in SIAR mixing model.**
(DOCX)

Table S4 **Pearson correlation analysis between trophic niche residuals after the LOWESS analysis and the prey biomass.**
(DOCX)
