# Supplementary material for: Seasonal Trophic Niche Shift and Cascading Effect of a Generalist Predator Fish
Source: PLoS One. 2012 Dec 14;7(12):e49691. doi: 10.1371/journal.pone.0049691 (PMC3522673; doi:10.1371/journal.pone.0049691)
Supplement: Table S1 — A Occurrence of food items in yellow catfish of different size in Lake Taihu. B. Dietary composition of yellow catfish in Lake Taihu. (DOCX) [file pone.0049691.s001.docx]

| Table S1A Occurrence of food items in yellow catfish of different size in Lake Taihu*. | | | | | | |
| --- | --- | --- | --- | --- | --- | --- |
| Food items | Size range (Fish length) | | | | | |
|  | 45mm-54mm | 55mm-64mm | 65mm-74mm | 75mm-99mm | 100mm-149mm | 140mm-249mm |
| Cladocera | 100.0 | 100.0 | 0.0 | 14.3 | 22.1 | 0.0 |
| Copepod | 100.0 | 66.7 | 66.7 | 14.3 | 0.0 | 0.0 |
| Clam and insect larva | 0.0 | 33.3 | 100.0 | 57.1 | 50.6 | 35.1 |
| Shrimp | 0.0 | 0.0 | 0.0 | 57.1 | 48.1 | 49.1 |
| Fishes | 0.0 | 0.0 | 0.0 | 14.3 | 32.2 | 38.6 |

| Table S1B Dietary composition of yellow catfish in Lake Taihu*. | | |
| --- | --- | --- |
| Species | Occurrence (%) | Percentage of number (%) |
| *Macrobrachium nipponense* | 21.5 | 13.5 |
| *Pseudorasbora parva* | 10.3 | 12.1 |
| *Rhodeus sp.* | 5.7 | 6.3 |
| *Hemiculter sp.* | 3.4 | 5.6 |
| *Parabramis sp.* | 0.5 | 0.9 |
| *Sarcocheilichthys sinensis sinensis* | 0.3 | 0.7 |
| *Macropodus ocellatus* | 0.3 | 2.0 |
| *Odontobutis obscurus* | 0.3 | 0.7 |
| Fish eggs | 2.3 | - |
| Unidentified fishes | 8.4 | 5.4 |
| Aquatic insects | 17.0 | 25.2 |
| Snails | 0.3 | 0.7 |
| Mussels | 20.5 | 26.2 |
| Sediment | 6.4 | - |

* Shexiao Zou and Beiping Tan. 1998. Studies on biology and fishery of *Pelteobagrus Fulvidraco* in Taihu Lake. Sichuan Institute of Animal Husbandry and Veterinary Medicine 12(1):36-41.
