# Supplementary material for: Seasonal Trophic Niche Shift and Cascading Effect of a Generalist Predator Fish
Source: PLoS One. 2012 Dec 14;7(12):e49691. doi: 10.1371/journal.pone.0049691 (PMC3522673; doi:10.1371/journal.pone.0049691)
Supplement: Table S2 — Seasonal variation in stable isotopic composition, weight and length of yellow catfish. (DOCX) [file pone.0049691.s002.docx]

Table S2. Seasonal variation in stable isotopic composition, weight and length of yellow catfish.

| Time | δ^15^N (‰) | SD | δ^13^C (‰) | SD | Weight | SD | Length | SD | N |
| --- | --- | --- | --- | --- | --- | --- | --- | --- | --- |
| April | 14.4 | 0.6 | -25.3 | 1.7 | 15.2 | 8.1 | 86.1 | 15.8 | 20 |
| May | 15.0 | 0.6 | -25.0 | 2.1 | 11.5 | 11.0 | 79.3 | 23.1 | 11 |
| June | 16.6 | 1.3 | -25.0 | 1.6 | 14.3 | 12.0 | 86.4 | 22.2 | 27 |
| July | 17.4 | 1.3 | -24.0 | 0.7 | 13.5 | 8.9 | 86.2 | 16.8 | 17 |
| August | 18.0 | 1.0 | -24.4 | 0.8 | 14.9 | 9.4 | 88.8 | 17.7 | 17 |
| September | 18.9 | 1.4 | -25.0 | 1.0 | 28.3 | 22.5 | 110.2 | 29.7 | 21 |
| October | 18.1 | 0.8 | -24.2 | 1.3 | 14.7 | 11.7 | 86.0 | 21.0 | 12 |
| November | 16.9 | 1.2 | -24.2 | 0.7 | 14.8 | 11.9 | 87.3 | 22.1 | 12 |
