# Supplementary material for: Seasonal Trophic Niche Shift and Cascading Effect of a Generalist Predator Fish
Source: PLoS One. 2012 Dec 14;7(12):e49691. doi: 10.1371/journal.pone.0049691 (PMC3522673; doi:10.1371/journal.pone.0049691)
Supplement: Table S3 — Seasonal variation in stable isotopic composition of yellow catfish prey used in SIAR mixing model. (DOCX) [file pone.0049691.s003.docx]

Table S3. Seasonal variation in stable isotopic composition of yellow catfish prey used in SIAR mixing model.

| Time | δ^15^N (‰) | | δ^13^C (‰) | | δ^15^N (‰) | | δ^13^C (‰) | | δ^15^N (‰) | | δ^13^C (‰) | |
| --- | --- | --- | --- | --- | --- | --- | --- | --- | --- | --- | --- | --- |
|  | E_pl_* | SD | E_pl_ * | SD | E_be_§ | SD | E_be_§ | SD | E_pi_† | SD | E_pi_† | SD |
| April | 9.6 | 0.2 | -28.5 | 0.4 | 11.8 | 0.6 | -22.5 | 0.4 | 14.8 | 0.5 | -24.5 | 0.6 |
| May | 11.1 | 0.4 | -28.9 | 0.7 | 12.2 | 0.3 | -22.4 | 0.5 | 17.3 | 0.6 | -24.5 | 0.9 |
| June | 10.7 | 0.5 | -28.4 | 1.2 | 12.6 | 0.5 | -22.5 | 0.7 | 17.4 | 0.4 | -25.8 | 0.4 |
| July | 11.7 | 0.5 | -27.6 | 0.5 | 13.3 | 0.4 | -22.2 | 0.2 | 16.7 | 0.1 | -24.3 | 0.6 |
| August | 11.6 | 0.4 | -27.4 | 0.6 | 13.0 | 0.4 | -22.4 | 0.4 | 17.6 | 0.9 | -24.6 | 0.6 |
| September | 12.7 | 0.2 | -27.3 | 0.3 | 13.2 | 0.4 | -22.4 | 0.9 | 17.7 | 0.5 | -26.2 | 1.0 |
| October | 10.7 | 0.4 | -27.5 | 0.5 | 10.8 | 0.8 | -21.4 | 0.3 | 17.5 | 0.2 | -24.4 | 0.7 |
| November | 10.6 | 0.3 | -27.3 | 0.2 | 11.6 | 0.7 | -22.2 | 0.3 | 17.4 | 0.4 | -25.1 | 0.4 |
| * E_pl_ represents the planktonic food sources, including zooplankton (n = 1) and mussels (*Cristaria plicata* and *Anodonta woodiana woodiana*) (n = 4) | | | | | | | | | | | | |
| §E_be_ represents the benthic food sources, including macrozoobenthos (n = 1) and snail (*Bellamya aeruginosa*) (n=4) | | | | | | | | | | | | |
| † E_pi_ represents the piscivorous food sources, including shrimps (*Exopalaemon modestus* and *Macrobrachium nipponensis*) and small fishes (*Pseudorasbora spp.*, *Rhinogobius spp.*, and *Odontobutis spp.*) (n=5) | | | | | | | | | | | | |
