# Supplementary material for: Seasonal Trophic Niche Shift and Cascading Effect of a Generalist Predator Fish
Source: PLoS One. 2012 Dec 14;7(12):e49691. doi: 10.1371/journal.pone.0049691 (PMC3522673; doi:10.1371/journal.pone.0049691)
Supplement: Table S4 — Pearson correlation analysis between trophic niche residuals after the LOWESS analysis and the prey biomass. (DOCX) [file pone.0049691.s004.docx]

| Table S4 Pearson correlation analysis between trophic niche residuals after the LOWESS analysis and the prey biomass. | | | |
| --- | --- | --- | --- |
| Residual types | zooplankton biomass | zoobenthos biomass | CPUE of fishes and shrimps |
| Planktivory residuals | -0.425^(*)^ | -0.179 | -0.791^**^ |
| Benthivory residuals | 0.336 | 0.136 | 0.081 |
| Piscivory residuals | 0.218 | 0.095 | 0.683^*^ |
| (*), * and ** represent significant relationships at 0.1 (marginally significant), 0.05 and 0.01 levels, respectively. | | | |
